# Transcriptomic profiling of the response to excess iodide in Keap1 hypomorphic mice reveals new gene-environment interactions in thyroid homeostasis

**DOI:** 10.1016/j.redox.2023.102978

**Published:** 2023-12-01

**Authors:** Panos G. Ziros, Dionysios V. Chartoumpekis, Ilias Georgakopoulos-Soares, Georgios Psarias, Gerasimos P. Sykiotis

**Affiliations:** a Service of Endocrinology, Diabetology and Metabolism, Lausanne University Hospital and University of Lausanne, 1011, Lausanne, Switzerland; b Department of Biochemistry and Molecular Biology, Penn State College of Medicine, Hershey, PA, USA

**Keywords:** Nrf2, Thyroid, Iodine, Proliferation, Antioxidant, Goiter

## Abstract

Iodide plays a pivotal role in thyroid homeostasis due to its crucial involvement in thyroid hormone biosynthesis. Exposure to pharmacological doses of iodide elicits in the thyroid an autoregulatory response to preserve thyroid function, as well as an antioxidant response that is mediated by the Keap1/Nrf2 signaling pathway. The objective of the present study was to investigate the transcriptional response of the thyroid to excess iodide in a background of enhanced Nrf2 signaling. Keap1 knockdown (Keap1^KD^) mice that have activated Nrf2 signaling were exposed or not to excess iodide in their drinking water for seven days and compared to respective wild-type mice. RNA-sequencing of individual mouse thyroids identified distinct transcriptomic patterns in response to iodide, with Keap1^KD^ mice showing an attenuated inflammatory response, altered thyroidal autoregulation, and enhanced cell growth/proliferative signaling, as confirmed also by Western blotting for key proteins involved in antioxidant, autoregulatory and proliferative responses. These findings underscore novel gene-environment interactions between the activation status of the Keap1/Nrf2 antioxidant response system and the dietary iodide intake, which may have implications not only for the goiter phenotype of Keap1^KD^ mice but also for humans harboring genetic variations in *KEAP1* or *NFE2L2* or treated with Nrf2-modulating drugs.

## Highlights

• Genetic activation of antioxidant signaling alters the thyroidal response to iodide overload.
• Mice with genetically activated Nrf2 showed an attenuated iodide-induced inflammatory response.
• They also showed altered thyroidal autoregulation and enhanced growth/proliferative signaling.

## Graphical abstract

**Figure:**
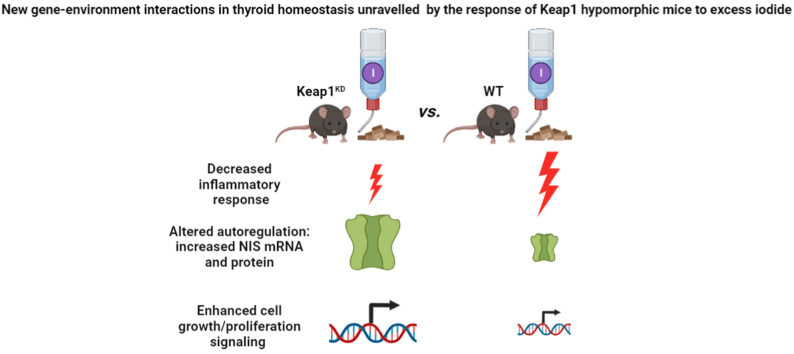

## 1 Introduction

Iodide plays a quintessential role in thyroid function and homeostasis, serving as a foundational element in thyroid hormone synthesis. The production of these hormones within the thyroid gland is contingent upon the availability of iodide. In a process marked by oxidative reactions, iodide (I^−^) undergoes oxidation to molecular iodine (I^0^) with the help of hydrogen peroxide generated locally. Subsequently, this oxidized iodine is incorporated into the structure of thyroglobulin (Tg), which is the principal protein of the thyroid gland [1]. In addition to processing physiological concentrations of dietary iodide for thyroid hormone synthesis, the normal thyroid gland is able to withstand exposure to pharmacological concentrations of iodide, such as those present in widely-used iodinated disinfectants or contrast media used in common radiological imaging like computed tomography (CT) scans or angiography. The ability to auto-regulate thyroid hormone synthesis in response to pharmacological iodide exposure in order to avoid iodide-induced hyper or hypothyroidism is called thyroid autoregulation [1]. The molecular mechanisms of thyroid autoregulation are generally not well defined, and the reasons why some individuals with thyroid disease, but also some with apparently healthy thyroids cannot tolerate excess iodide are poorly known [2].

Our previous investigations demonstrated an activation of the Keap1/Nrf2 cytoprotective pathway in response to pharmacological concentrations of iodide in wild-type (WT) mice [3,4], as well as aspects of altered thyroid autoregulation in Nrf2 knockout (KO) mice [3]. Nrf2, a transcription factor, is ordinarily maintained in the cytoplasm by Keap1, which facilitates its degradation by the proteasome under baseline conditions. However, exposure to reactive oxygen species (ROS) triggers the oxidation of specific sulfhydryl groups in Keap1 cysteines, which induces a conformational change in Keap1 that incapacitates its ability to target Nrf2 for degradation. Nrf2 can also be activated pharmacologically by various natural or synthetic compounds [[5], [6], [7], [8]]. Consequently, Nrf2 that has been newly transcribed and translated accumulates and migrates into the nucleus, where it binds to specific antioxidant response element (ARE) DNA sequences located in the regulatory regions of its target genes resulting in their upregulation [9]. Our previous work on the thyroid has shown that, in addition to antioxidant and other cytoprotective genes, Nrf2 directly upregulates the transcription of the gene encoding Tg via two AREs in its upstream distal enhancer [3].

Mice with deletion of Nrf2, despite reduced *Tg* mRNA expression and Tg protein abundance in the thyroid, show higher levels of Tg iodination at baseline. This increases further in response to pharmacological iodide exposure, in contrast to the physiological decrease that is observed in wild-type mice [3]. In addition, Nrf2 deletion potentiates the iodide-induced inflammatory and immune transcriptional response in the thyroid [4]. However, there are no studies describing the effect of iodide on the thyroid in the presence of an activated Nrf2 pathway. We have previously shown that mice exhibiting enhanced Nrf2 function due to Keap1 hypomorphism show higher expression of antioxidant genes and increased degradation of Tg, and they develop a goiter (thyroid enlargement) phenotype that is characterized by increased thyroid follicle size, thyroid weight and TSH levels, indicating the presence of subclinical hypothyroidism [10]. The objective of the present study was to characterize the thyroid transcriptomic response of Keap1 hypomorphic mice to a pharmacological dose of iodide. The results indicate a novel gene-environment functional interaction, whereby iodide exposure and Nrf2 activation status jointly determine the thyroid follicular cells’ inflammatory, autoregulatory, proliferative and growth transcriptional responses.

## 2 Materials and methods

### 2.1 Mice

C57BL/6 J *Keap1*^*flox/flox*^ mice were developed by Prof. Masayuki Yamamoto [11]. These mice express lower levels of Keap1 because of the loxP site insertions [12] and are designated as Keap1^KD^. 3–4 months old WT and Keap1^KD^ mice fed a standard diet (KLIBA NAFAG 3242, Switzerland), containing 1.5 mg/kg sodium iodide, were given normal tap water with or without 0.05 % sodium iodide (Sigma, St Louis, MO, USA) for 7 days. Hence, the following groups of mice were included: WT mice on control water (n = 8), WT mice exposed to excess iodide (WT-IOD, n = 6), Keap1^KD^ mice on control water (n = 8), and Keap1^KD^ mice exposed to excess iodide (Keap1^KD^-IOD, n = 6). Mice were maintained in the animal facility of the Department of Physiology at the University of Lausanne in temperature-, light-, and humidity-controlled rooms with a 12-h light/dark cycle. At the end of the experiment, mice were euthanized; thyroid and pituitary tissue were harvested and stored in RNAlater® (ThermoFischer Scientific, Ecublens, Switzerland), and plasma was prepared and frozen at −80 °C. All animal procedures were in accordance with Swiss legislature and the study was approved by the Canton of Vaud SCAV.

### 2.2 Next-generation messenger RNA (mRNA) sequencing

RNA from individual mouse thyroids was prepared as previously described [13] and was submitted to Alithea Genomics (Switzerland). The bulk RNA barcoding and sequencing (BRB-seq) libraries were generated and sequenced as described previously [14] to a depth of approximately 1.2 million raw reads per sample. Sequencing data were uploaded to Zenodo (https://zenodo.org/record/8382691). The iDEP platform [15] was used to pipeline the gene expression analysis. To eliminate genes with low expression, minimum CPM (counts per million reads) was set to 1 for all samples. edgeR [16] was used to transform count data for clustering and PCA analysis. DESeq2 [17] was employed to analyze the differential expression of genes with a minimum fold-change (increase or decrease) of 1.5 and with a false discovery rate (FDR) cutoff set at <0.05. Pathway analysis of the differentially expressed genes was performed using parametric analysis of gene set enrichment (PGSEA) embedded in the iDEP platform, and by use of the Ingenuity Pathway Analysis (IPA) platform (QIAGEN Inc.) [18].

### 2.3 Real-time PCR

Real-time PCR was performed as previously reported using primers and conditions previously described [3].

### 2.4 Western immunoblotting

Total protein isolation from individual mouse thyroids was performed as previous described [13]; Western immunoblotting was performed using the antibodies listed in our previous work [10] and a Ki67 antibody from Abcam (ab16667, dilution 1:500).

### 2.5 Statistics

For calling differentially expressed genes, the statistical testing methods employed were those embedded in the DESeq 2 suite. Two-way ANOVA was used for the comparison of real-time PCR-based relative gene expression. For other analyses, specific details are provided in the relevant figure legends. Statistical significance was set at p < 0.05 for all analyses.

## 3 Results and discussion

Transcriptomic profiling of WT and Keap1^KD^ mice identified distinct gene clusters of differentially expressed genes and enriched pathways depending on genotype and treatment.

**Fig. 1:**
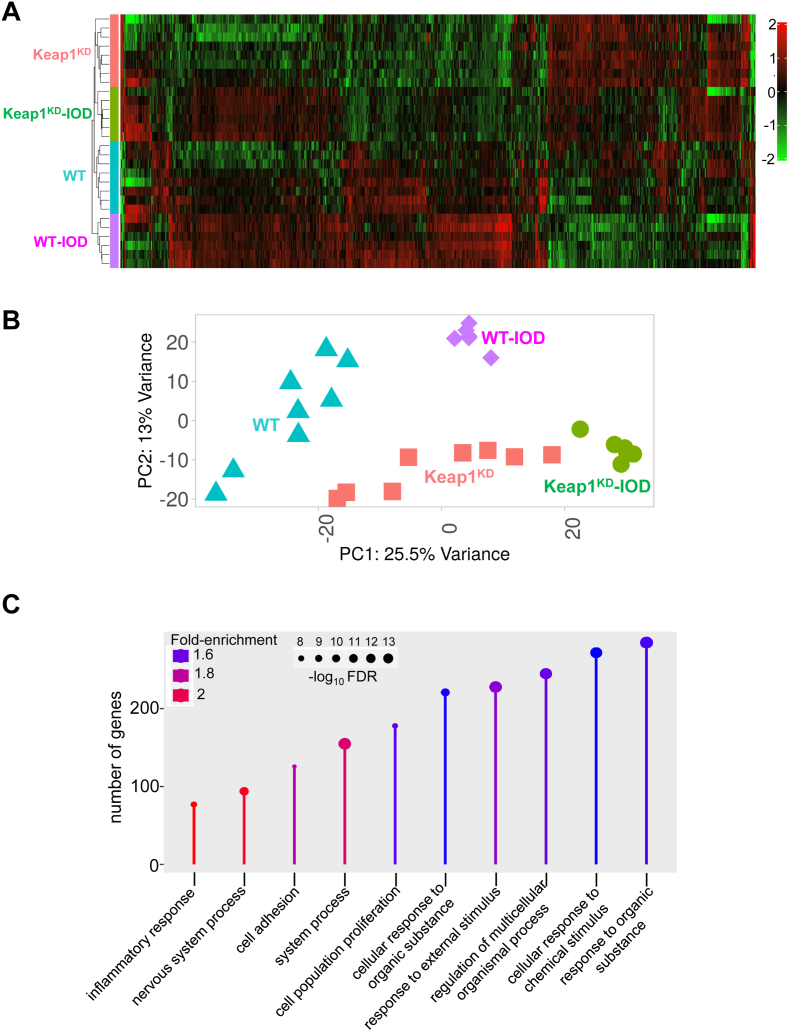
**A.** Heatmap and clustering of the 1000 genes showing the most variable expression in the thyroid. The heatmap was clustered by Euclidean distancing and average linkage. Red color indicates higher expression and green color lower expression. **B.** Principal component analysis (PCA) of RNA-Seq data. Of the total variance in gene expression, 25.5 % can be attributed to PC1, and 13 % can be attributed to PC2. **C.** Lollipop plot showing the ten most highly enriched GO biological processes in the 1000 most variable genes in the dataset. WT: wild-type mice; Keap1^KD^: Keap1 hypomorphic mice; IOD: mice exposed to excess iodide in their drinking water (NaI, 0.05 %). (For interpretation of the references to color in this figure legend, the reader is referred to the Web version of this article.)

The most highly variable genes in the thyroids of all mice clustered perfectly by genotype and treatment (Fig. 1A). The Principal Component Analysis (PCA) further supports this observation, demonstrating that the distinct mouse groups cluster according to two primary principal components. The first one (PC1), represented on the horizontal axis, accounts for 25.5 % of the variance in gene expression; the second (PC2), represented on the vertical axis, accounts for 13 % of the variance (Fig. 1B). A gene ontology analysis of the 1000 genes with the most variable expression showed a high enrichment for functions including response to external stimuli and stressors, inflammatory response, and cell proliferation (Fig. 1C).

**Fig. 2:**
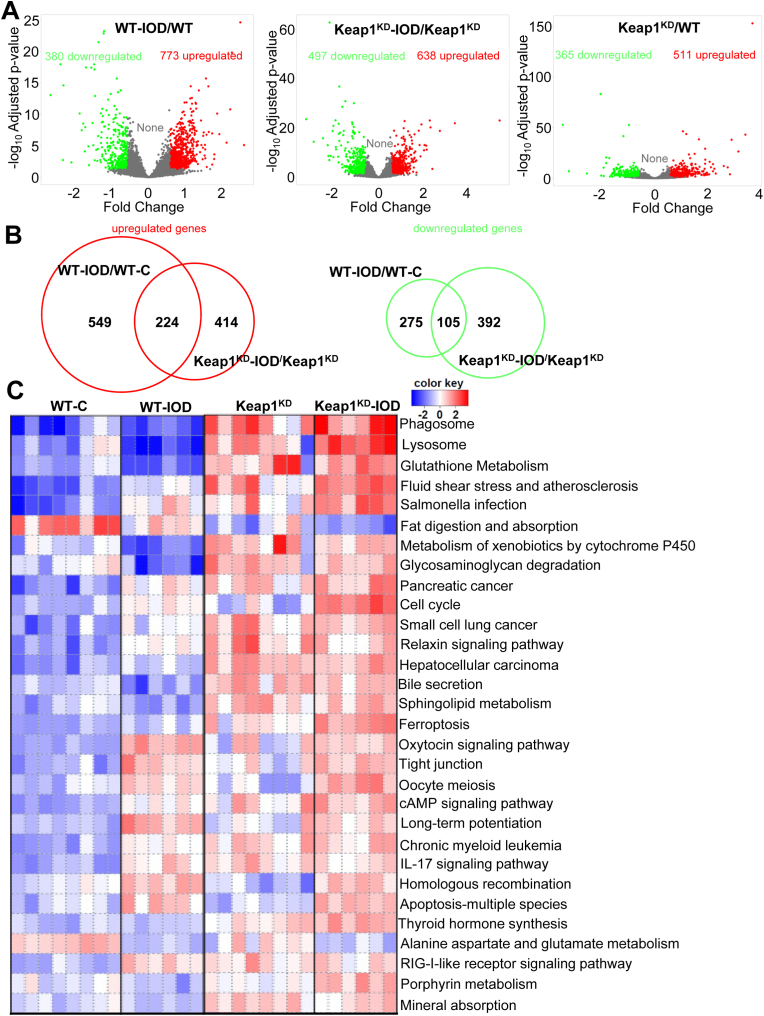
**A.** Volcano plots of differentially expressed genes (FDR<0.05, fold change>1.5) in iodide-treated WT mice (WT-IOD vs. WT), in iodide-treated Keap1^KD^ mice (Keap1^KD^-IOD vs. Keap1^KD^), and in the two genotypes at baseline (Keap1^KD^ vs. WT). **B.** Venn diagrams depicting the number of unique and overlapping up- or down-regulated genes in WT vs. Keap1^KD^ mice after iodide treatment. **C.** Pathway analysis of all of the aforementioned differentially expressed genes using PGSEA. The heatmap depicts the trend for each KEGG pathway for each sample. Red squares indicate upregulation (positive z-score), while blue squares indicate downregulation (negative z-score). The top 30 pathways are shown, with FDR set at <0.05. (For interpretation of the references to color in this figure legend, the reader is referred to the Web version of this article.)

Iodide increased the expression of 773 genes in WT and 638 in Keap1^KD^ mice at by least 1.5-fold (Fig. 2A), with 224 genes being common among the two genotypes (Fig. 2B). 380 and 497 genes in WT and Keap1^KD^ mice, respectively, showed decreased expression by at least 1.5-fold after iodide exposure (Fig. 2A) with 105 being common (Fig. 2B). At baseline, 511 genes are upregulated and 365 downregulated at least 1.5-fold in Keap1^KD^ mice compared to WT (Fig. 2A). Among the most enriched pathways identified by pathway analysis performed on the differentially expressed genes were the following: glutathione and xenobiotic metabolism; thyroid hormone synthesis; phagosome and lysosome functions; and cell proliferation (Fig. 2C). Each of these is further discussed separately below.

### 3.1 Antioxidant response

**Fig. 3:**
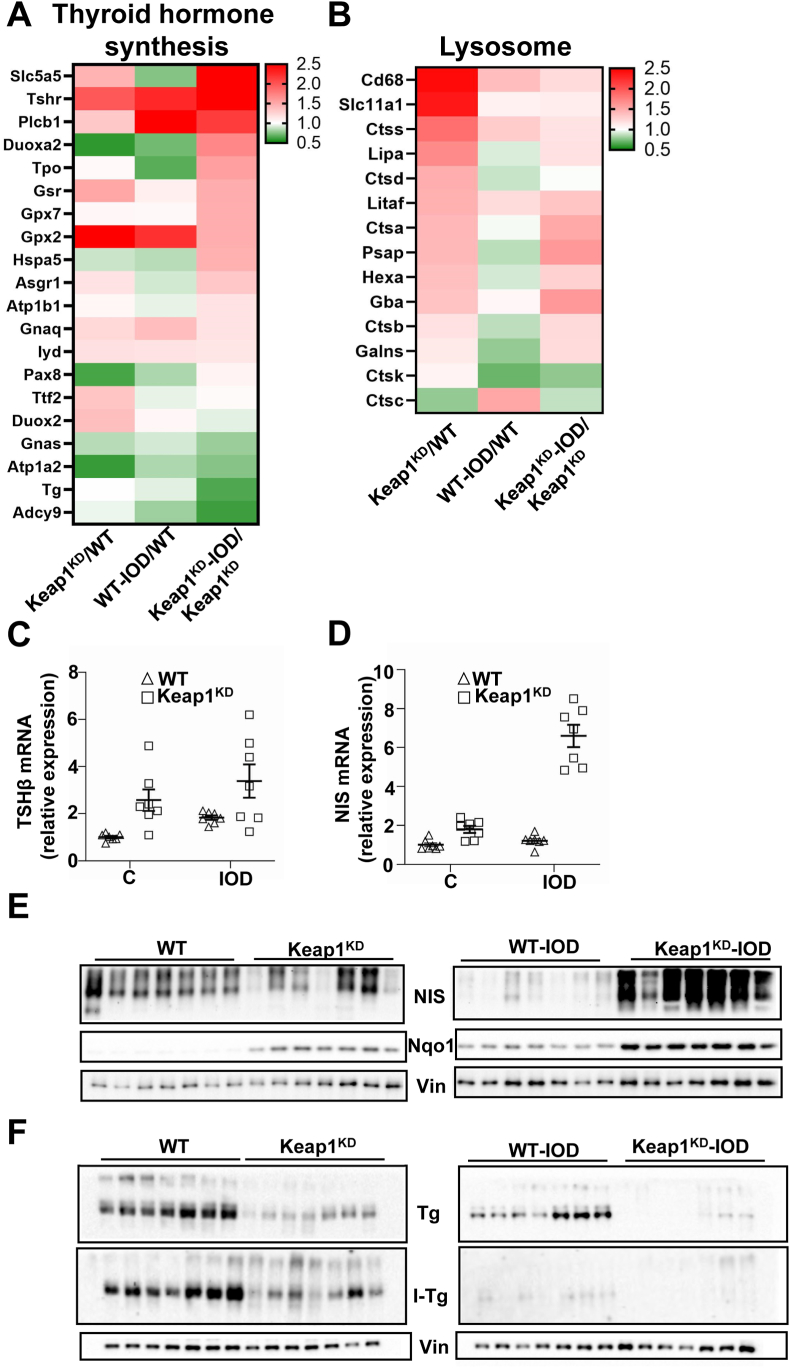
Heatmaps showing the fold-change in the expression of highlighted genes in enriched pathways from Fig. 2C: **A.** thyroid hormone synthesis and **B.** lysosome. **C.** TSHβ mRNA expression levels in the pituitary gland assessed by real-time PCR. Two-way ANOVA statistical analysis showed a significant (p < 0.05) effect of genotype and non significant (ns) effects of treatment and genotype-treatment interaction. C: control; IOD: iodide. **D.** Sodium-iodide symporter (NIS) mRNA expression levels in the thyroid assessed by real-time PCR. Two-way ANOVA showed significant (p < 0.05) effects of genotype, treatment and their interaction. **E.** NIS and Nqo1 protein abundance by immunoblotting in thyroids of WT and Keap1^KD^ mice exposed or not to excess iodide. Vinculin (Vin) is used as a loading control. **F.** Thyroglobulin (Tg) and iodinated thyroglobulin (I-Tg) abundance by immunoblotting in thyroids of WT and Keap1^KD^ mice exposed or not to excess iodide. Vinculin (Vin) is used as a loading control. This is a separate blot from the one in panel E.

**Supplemental Fig. 1:**
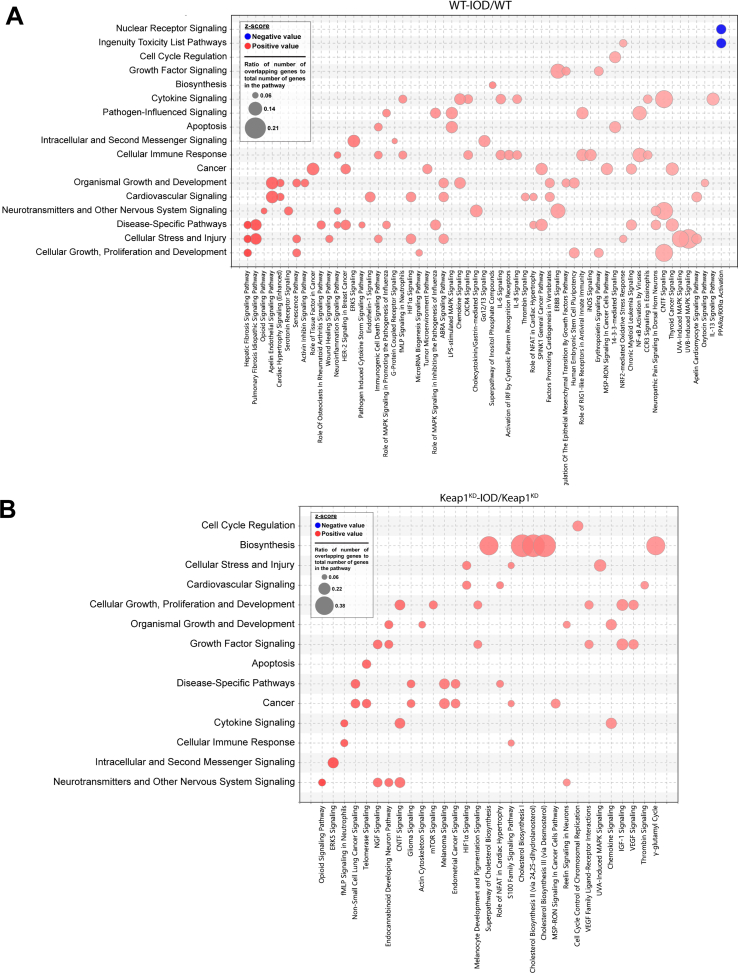
Bubble chart generated by Ingenuity Pathway Analysis (IPA) and showing enriched pathways according to genes that show differential expression after iodide exposure of **A.** WT mice (WT-IOD vs. WT) and **B.** Keap1^KD^ mice (Keap1^KD^-IOD vs. Keap1^KD^). The Y axis represents pathway categories and the X axis shows the actual individual pathways. The z-score represents the activation or inhibition state of a canonical pathway. Only pathways with an absolute z-score>2 are depicted and ranked by z-score.

**Supplemental Fig. 2:**
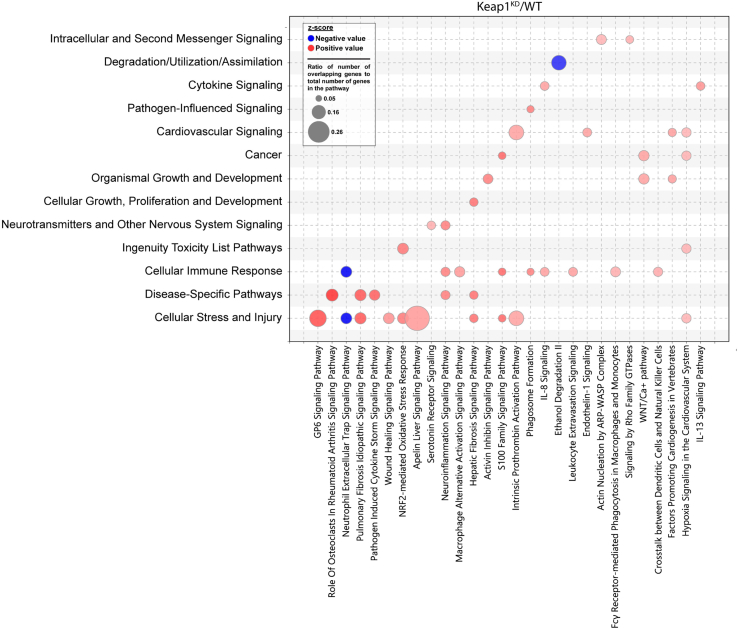
Bubble chart generated by IPA and showing enriched pathways according to genes that show differential expression between Keap1^KD^ and WT mice without excess iodide. The Y axis represents pathway categories and the X axis shows the actual individual pathways. Only pathways with an absolute z-score > 2 are depicted and ranked by z-score.

**Supplemental Fig. 3:**
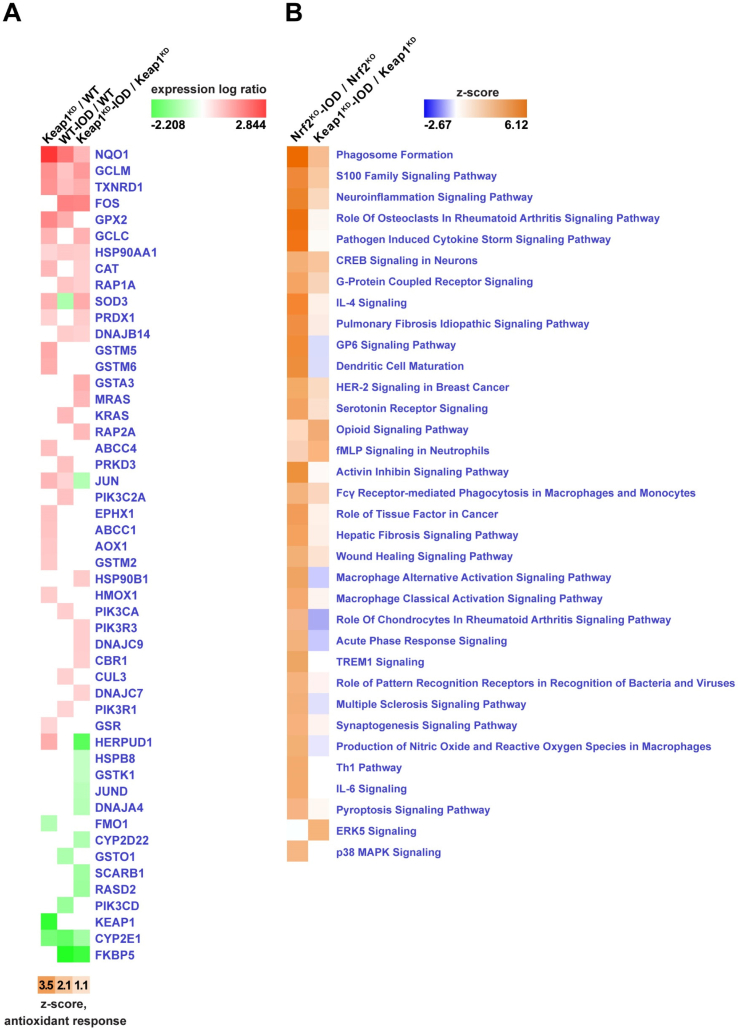
**A**. Heatmap generated by IPA showing the relative expression of genes that participate in the “Nrf2-mediated oxidative stress response” pathway, shown as “antioxidant response” in the figure for brevity. **B.** Comparative analysis by IPA of pathways enriched in Keap1^KD^ mice after iodide exposure (present work) versus pathways enriched after iodide exposure in Nrf2^KO^ mice in our previous work [4]. Pathways with an absolute z-score > 3 are shown.

**Supplemental Fig. 4:**
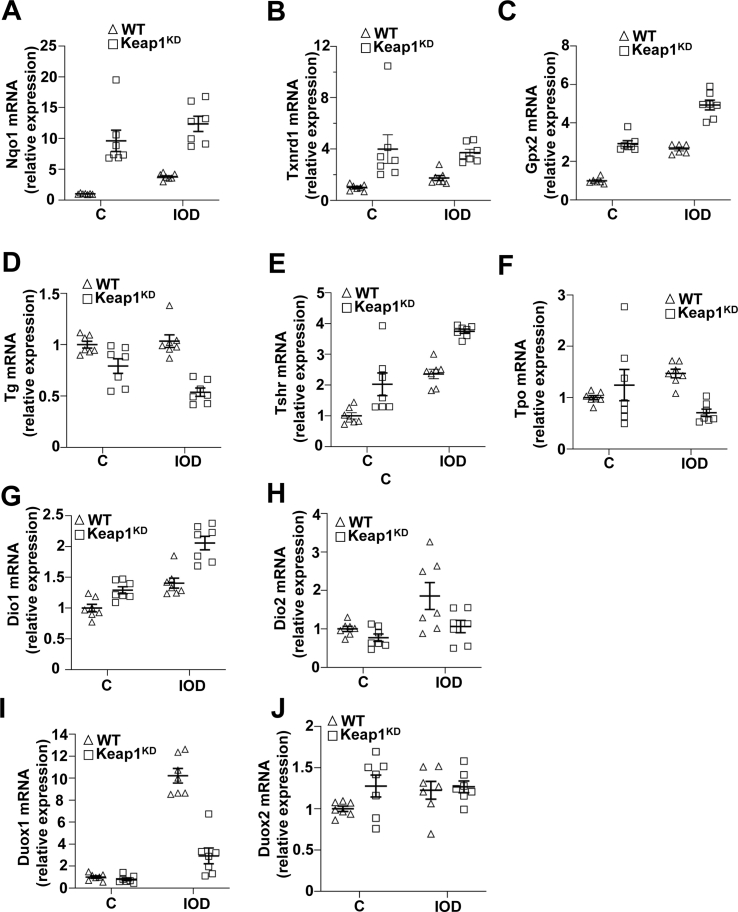
Real-time PCR-based relative gene expression analyses of selected genes in the thyroid. The main effects of genotype and treatment and the genotype-treatment interaction effect were tested by two-way ANOVA, yielding the following results for each gene: **A.** Nqo1; interaction: ns, treatment: p < 0.05, genotype: p < 0.05. **B.** Txnrd1; interaction: ns, treatment: ns, genotype: p < 0.05. **C.** Gpx2; interaction: ns, treatment: p < 0.05, genotype: p < 0.05. **D.** Tg; interaction: p < 0.05, treatment: ns, genotype: p < 0.05. **E.** Tshr; interaction: ns, treatment: p < 0.05, genotype: p < 0.05. **F.** Tpo; interaction: p < 0.05, treatment: ns, genotype: ns. **G.** Dio1; interaction: p < 0.05, treatment: p < 0.05, genotype: p < 0.05. **H.** Dio2; interaction: ns, treatment: p < 0.05, genotype: p < 0.05. **I.** Duox1; interaction: p < 0.05, treatment: p < 0.05, genotype: p < 0.05. **J.** Duox2; interaction: ns, treatment: ns, genotype: ns. C: control; IOD: iodide.

Genes related to glutathione and xenobiotic metabolism, as well as to ferroptosis, were upregulated in Keap1^KD^ mice, as reflected in both PGSEA (Fig. 2C) and IPA profiling (Supp. Fig. 2). This is expected, because Nrf2 is a master regulator of antioxidant/cytoprotective genes (Supp. Fig. 2) and has been recently shown to control the expression of genes that prevent lipid peroxidation and ferroptosis [19]. Without excess iodide, activation of the Nrf2 pathway (z-score = 3.5) by Keap1 knockdown leads to the induction of cytoprotective Nrf2 target genes such as Nqo1, Gpx2, Txnrd1 and Gclc (Supp. Fig. 3A, Supp. Figs. 4A–C and Fig. 3E). The Nrf2 pathway is also induced by exposure to iodide in WT mice (z-score = 2.1) (Supp. Fig. 1A, Supp. Fig. 3A), consistent with our previous findings [3,4]; this is also reflected in the protein abundance of Nqo1 (Fig. 3E). Exposure of Keap1^KD^ mice to iodide further induces the Nrf2 pathway, but to a lesser extent (z-score = 1.1) (Supp. Fig. 3A), likely reflecting a limited capacity to further stimulate a pathway that is already highly active.

In our previous work we demonstrated that exposure of mice lacking Nrf2 to excess iodide led to higher upregulation of pathways related to inflammation and immune response compared to WT mice [4]. The treatment, age and sex of the mice were similar between that experiment and the present one, but the mouse facilities and the methods of RNA sequencing were different, precluding a direct comparison of differential gene expression between these two experiments. Therefore, we performed a qualitative analysis to compare the pathways that are enriched after iodide exposure in Nrf2^KO^ and Keap1^KD^ mice. Interestingly, several pathways related to inflammation and immune response were much more highly enriched and upregulated in the Nrf2^KO^ mice after iodide exposure compared to the Keap1^KD^ mice (S100 signaling pathway, neuroinflammation, IL-4 signaling, pathogen-induced cytokine response, GP6 signaling, Th1 pathway, IL-6 signaling, etc.) (Supp. Fig. 3B). These findings further confirm the protective effect of Nrf2 against an iodide-induced inflammatory response in the thyroid.

### 3.2 Thyroid hormone synthesis, phagosome and lysosome functions

Pathways containing genes related to thyroid hormone synthesis (Fig. 2, Fig. 3A) or to lysosomal function (Fig. 2, Fig. 3B) were also significantly enriched; the expression of individual thyroid hormone synthesis-related genes was also tested by RT-PCR (Supp. Figs. 4D–J). We therefore compared the pituitary mRNA expression levels of β-TSH (measured by RT-PCR as a highly sensitive marker of thyroid function [20] by two-way ANOVA; the results showed a significant effect of the genotype (p = 0.011), a near-significant effect of iodide treatment (p = 0.066), and no interaction effect between genotype and treatment (p = 0.98), indicating that the Keap1 genotype does not modify the effect of iodide overload on thyroid function (Fig. 3C). Nevertheless, similar analysis of the thyroidal mRNA expression levels of the sodium-iodide symporter (NIS, encoded by the *Slc5a5* gene) that facilitates the active transport of iodide into thyroid follicular cells showed significant effects of the genotype (p < 0.0001), the treatment (p < 0.0001), and their interaction (p < 0.0001), with markedly higher NIS expression levels in Keap1^KD^ mice after iodide exposure (Fig. 3D). At the protein level, NIS protein abundance decreased in response to iodide in WT mice, but it markedly increased in iodide-treated Keap1^KD^ mice (Fig. 3E). Regarding Tg, the abundance of the total protein was lower in Keap1^KD^ mice compared to WT mice (Fig. 3F), possibly related to altered lysosomal function (Fig. 3B), including altered expression of Tg-processing cathepsins, as characterized also in our previous work [10]. The abundance of the iodinated form of Tg (I-Tg) was also lower in Keap1^KD^ mice compared to WT mice, and the reduction in the abundance of both forms in iodide-treated WT mice was observed in Keap1^KD^ mice as well (Fig. 3F). Taken together, these results indicate that thyroidal autoregulation in response to iodide overload is partially altered in Keap1^KD^ mice, with a paradoxical increase in NIS but a physiological Tg and I-Tg response pattern. Interestingly, this is the opposite of what we had previously found in Nrf2^KO^ mice, where the NIS response pattern following iodide overload was preserved, whereas I-Tg showed a paradoxical increase (instead of the expected physiological decrease) [3]. In conclusion, Nrf2 pathway activity impacts thyroid autoregulation in conditions of iodide overload, with different response patterns in Nrf2-gain vs. -loss-of-function conditions.

### 3.3 Cell growth/proliferation

**Fig. 4:**
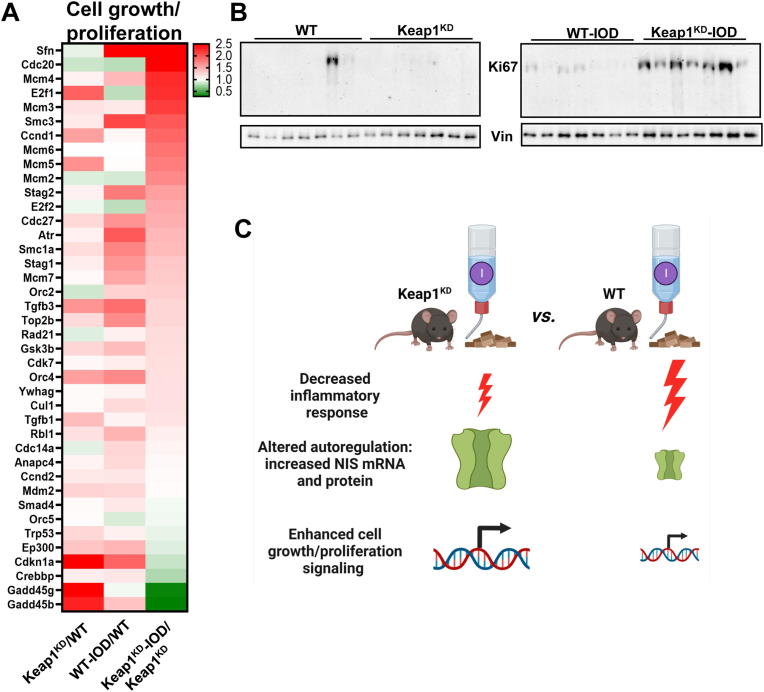
**A.** Heatmap showing the fold-change in the expression of highlighted genes participating in cell growth/proliferation pathways. **B.** Ki67 protein abundance by immunoblotting in thyroids of WT and Keap1^KD^ mice exposed or not to excess iodide. Vinculin (Vin) is used as a loading control. This is the same blot as in Fig. 3E (same loading control shown). **C.** Model summarizing the results of the present study.

As mentioned, we previously demonstrated that Keap1^KD^ mice develop goiter whose size increases with age, alongside with higher pituitary and plasma levels of TSH, indicative of compensated hypothyroidism [10]. In the present work, pathway analysis of the differentially expressed genes among the mice identified a significant enrichment of cell growth/proliferation pathways (reflected by the terms “pancreatic cancer”, “cell cycle”, “small cell lung cancer”, “hepatocellular carcinoma”, “chronic myeloid leukemia”, etc.) (Fig. 2C). Overall, these cell growth/proliferation-related pathways tend to show a similar pattern, with upregulation in Keap1^KD^ versus WT mice at baseline, and with further increase upon iodide exposure mainly in Keap1^KD^ mice (Fig. 2C). The overrepresentation of cell growth/proliferation pathways was corroborated by separate IPA profiling for each pair-wise comparison among the different mouse groups (Supp. Fig. 1). The aforementioned pattern showing further induction in iodide-treated Keap1^KD^ mice is also evident in several individual cell growth/proliferation-related genes that are listed in Fig. 4A together with their relative fold-changes. This is also confirmed at the protein level, with Ki67 protein abundance following a similar pattern (Fig. 4B). In previous work, we have characterized by histomorphometry the goiter phenotype of Keap1^KD^ mice at baseline conditions [10]. The markedly increased follicle area corresponded primarily to a markedly increased colloid area; nevertheless, thyrocyte area and thyrocyte size (per follicle) were increased, whereas thyrocyte number (per follicle) was similar [10]. These observations indicate that cell growth likely contributes to the goiter phenotype of Keap1^KD^ mice. In the present work, Keap1^KD^ mice showed upregulation of not only growth-related but also proliferation-related pathways both at baseline (Fig. 2C and Supp. Fig. 2) and in response to short-term iodide overload (Fig. 2C).

## 4 Conclusions

To summarize, when exposed to iodide overload, mice with genetically activated Nrf2 signaling show an attenuated iodide-induced inflammatory response in the thyroid, altered thyroidal autoregulation with a paradoxical increase in NIS, and enhanced activation of cell growth- and proliferation-related pathways compared to WT mice (Fig. 4C). These findings highlight new gene-environment interactions between the genetic status of the Keap1/Nrf2 antioxidant response system and the dietary iodide intake, and they contribute to our understanding of the complex mechanisms underlying redox balance and imbalance, including Nrf2 signaling [21]. These interactions may have implications for the phenotype of mouse models with altered Nrf2 signaling (e.g., the goiter phenotype of Keap1^KD^ mice). In terms of clinical implications, they may have relevance for humans with functional genetic variation in *NFE2L2* [22] or *KEAP1* [23] or under treatment with clinically approved NRF2 modulating drugs (i.e., dimethyl fumarate [24] and ovameloxolone [25]); it will be relevant to examine whether individuals with genetically or pharmacologically activated Nrf2 signaling are more susceptible to thyroid diseases such as goiter.

## Author contributions

P.G.Z. and G.P.S. designed the study; P.G.Z., D.V·C., I.G.-S., G.P. and G.P.S. performed experiments; P.G.Z., D.V.C. and G.P.S. drafted the article; and all authors analyzed data and edited the article.

## Declaration of competing interest

None of the authors has a competing interest to declare.
