# Supplementary material for: Transcriptomic profiling of the response to excess iodide in Keap1 hypomorphic mice reveals new gene-environment interactions in thyroid homeostasis
Source: Redox Biol. 2023 Dec 1;69:102978. doi: 10.1016/j.redox.2023.102978 (PMC10746517; doi:10.1016/j.redox.2023.102978)
Supplement: Multimedia component 1 [file mmc1.docx]

**Supplemental Figure 1.** Bubble chart generated by Ingenuity Pathway Analysis (IPA) and showing enriched pathways according to genes that show differential expression after iodide exposure of **A.** WT mice (WT-IOD vs. WT) and **B.** Keap1^KD^ mice (Keap1^KD^-IOD vs. Keap1^KD^). The Y axis represents pathway categories and the X axis shows the actual individual pathways. The z-score represents the activation or inhibition state of a canonical pathway. Only pathways with an absolute z-score>2 are depicted and ranked by z-score.

**Supplemental Figure 2.** Bubble chart generated by IPA and showing enriched pathways according to genes that show differential expression between Keap1^KD^ and WT mice without excess iodide. The Y axis represents pathway categories and the X axis shows the actual individual pathways. Only pathways with an absolute z-score>2 are depicted and ranked by z-score.

**Supplemental Figure 3. A.** Heatmap generated by IPA showing the relative expression of genes that participate in the “Nrf2-mediated oxidative stress response” pathway, shown as “antioxidant response” in the figure for the sake of brevity. **B.** Comparative analysis by IPA of pathways enriched in Keap1^KD^ mice after iodide exposure (present work) versus pathways enriched after iodide exposure in Nrf2^KO^ mice in our previous work [4]. Pathways with an absolute z-score>3 are shown.

**Supplemental Figure 4.** Real-time PCR-based relative gene expression analyses of selected genes in the thyroid. The main effects of genotype and treatment and the genotype-treatment interaction effect were tested by wo-way ANOVA, yielding the following results for each gene: **A.** Nqo1; interaction: ns, treatment: p<0.05, genotype: p<0.05. **B.** Txnrd1; interaction: ns, treatment: ns, genotype: p<0.05. **C.** Gpx2; interaction: ns, treatment: p<0.05, genotype: p<0.05. **D.** Tg; interaction: p<0.05, treatment: ns, genotype: p<0.05. **E.** TSHR; interaction: ns, treatment: p<0.05, genotype: p<0.05. **F.** TPO; interaction: p<0.05, treatment: ns, genotype: ns. **G.** Dio1; interaction: p<0.05, treatment: p<0.05, genotype: p<0.05. **H.** Dio2; interaction: ns, treatment: p<0.05, genotype: p<0.05. **I.** Duox1; interaction: p<0.05, treatment: p<0.05, genotype: p<0.05. **J.** Duox2; interaction: ns, treatment: ns, genotype: ns. C: control; IOD: iodide.
